# Recent Advances in the Analysis of Macromolecular Interactions Using the Matrix-Free Method of Sedimentation in the Analytical Ultracentrifuge

**DOI:** 10.3390/biology4010237

**Published:** 2015-03-06

**Authors:** Stephen E. Harding, Richard B. Gillis, Fahad Almutairi, Tayyibe Erten, M. Şamil Kök, Gary G. Adams

**Affiliations:** 1 National Centre for Macromolecular Hydrodynamics, University of Nottingham, Sutton Bonington LE12 5RD, UK; E-Mails: stxrbg@nottingham.ac.uk (R.B.G.); stxfa7@nottingham.ac.uk (F.A.); stxte@nottingham.ac.uk (T.E.); gary.adams@nottingham.ac.uk (G.G.A.); 2 Department of Food Engineering, Abant Izzet Baysal University, Bolu 14280, Turkey; E-Mail: kok_s@ibu.edu.tr; 3 Faculty of Medicine and Health Sciences, University of Nottingham, Clifton Boulevard, Nottingham NG7 2RD, UK

**Keywords:** protein, carbohydrate, nucleic acid, interaction, hydrodynamics

## Abstract

Sedimentation in the analytical ultracentrifuge is a matrix free solution technique with no immobilisation, columns, or membranes required and can be used to study self-association and complex or “hetero”-interactions, stoichiometry, reversibility and interaction strength of a wide variety of macromolecular types and across a very large dynamic range (dissociation constants from 10^−12^ M to 10^−1^ M). We extend an earlier review specifically highlighting advances in sedimentation velocity and sedimentation equilibrium in the analytical ultracentrifuge applied to protein interactions and mucoadhesion and to review recent applications in protein self-association (tetanus toxoid, agrin), protein-like carbohydrate association (aminocelluloses), carbohydrate-protein interactions (polysaccharide-gliadin), nucleic-acid protein (G-duplexes), nucleic acid-carbohydrate (DNA-chitosan) and finally carbohydrate-carbohydrate (xanthan-chitosan and a ternary polysaccharide complex) interactions.

## 1. Introduction

We recently [1] gave a detailed consideration of how the Analytical Ultracentrifuge—a matrix-free separation technique for the analysis of the concentration distributions of a protein solute in solution at high centrifugal field—can be used to investigate either how this concentration distribution changes with time (known as sedimentation velocity), or (at relatively lower centrifugal fields using the same instrumentation) the final steady state concentration distribution after sedimentation and diffusive forces have come to equilibrium (sedimentation equilibrium), can be applied to the study of protein interactions. The choice of the appropriate optical system was shown to be important, with sometimes combined optics (refractometic and UV absorption) proving useful via the process of co-sedimentation, especially when used to study mucoadhesive interactions for drug delivery applications [2,3], and protein-cofactor binding [1].

Since that time there have been some important developments in terms of analysis, particularly for the sedimentation velocity and equilibrium analysis of heterogeneous materials. We will briefly review those developments and then consider very recent applications to protein self-association (tetanus toxoid, the yeast exoribonuclease co-factor Rrp47 and mini-agrin), protein-like carbohydrate self-association (aminocelluloses), carbohydrate-protein interactions (polysaccharide-gliadin), nucleic acid-carbohydrate (DNA-chitosan), carbohydrate-carbohydrate (xanthan-chitosan and PGX^®^ complex) interactions and nucleic acid-protein interactions (G-quadruplex—RNA helicase).

## 2. Analytical Ultracentrifugation

The analytical ultracentrifuge is a high speed (to 60,000 rpm) ultracentrifuge with one or more optical systems to allow the analysis of the redistribution of macromolecular solute under the influence of a centrifugal field [4,5]. This can be either a sedimentation velocity experiment where the change in concentration distribution with time is followed, or a sedimentation equilibrium experiment—performed at lower speeds—where the steady state distribution of the macromolecular solute is recorded following equilibration of centrifugal and diffusive forces. The following are common types of optical systems used to register concentration distributions: (i) UV/visible—useful for proteins and nucleic acids which generally absorb in the UV, and coloured macromolecules like the cytochromes which absorb in the visible; (ii) refractometric or interferometric optics, which can be used for any macromolecular system. Two other types of optical system are used; (iii) fluorescence optics, permitting detection at very low concentration; and (iv) refractive index gradient or “Schlieren” optics, available on some older equipment.

## 3. Sedimentation Velocity

In sedimentation velocity we measure the sedimentation coefficient distribution *g(s)* *vs.* *s*—a measure of the purity/heterogeneity of the sample. The *s* value of individual components present can be related to the molecular weight and (from the frictional properties) shape. The greater the molecular weight or more compact the macromolecule is, the larger its *s* value. It is often converted to standard conditions (the density and viscosity of water at 20.0 °C)—to give *s*_20,w_. Modern software, such as the highly popular SEDFIT suite of algorithms of Schuck and colleagues [6,7] provides also:

(i) A correction to the sedimentation coefficient distribution for diffusion broadening (this assumes all particles in the distribution have the same frictional ratio *f*/*f_o_* (ratio of the friction coefficient of a macromolecule to the friction coefficient of a sphere of the same mass and anhydrous volume)—the resulting corrected distribution is known as a *c(s) vs.* *s* distribution.
(ii) Conversion of *g(s)* *vs.* *s* or *c(s) vs.* *s* for a *discrete* distribution (*i.e.*, of a small number of resolvable components) to a molecular weight distribution (again, assuming particles of the same shape/frictional ratio), known as *g(M)* *vs.* *M* or *c(M)* *vs.* *M.*
(iii) For continuous distributions, a recently published algorithm known as the *Extended Fujita* algorithm [8] was incorporated into SEDFIT.

The transformation in the Extended Fujita algorithm is as follows:

(1) f(M)=(ds/dM.gs)

with

(2) $$M={\left(s/\kappa_{s}\right)}^{1/b}$$

and

(3) $$ds/dM=b.{\kappa_{s}}^{1/b}.s^{(b-1)/b}$$

*b* is a conformation parameter (~2/3 for globular macromolecules, decreasing for more asymmetric structures down to ~0.15 for rigid rods). κ*_s_* can be found from Equation (2) provided that at least one value of *M* (e.g., *M*_w_ from sedimentation equilibrium) is known for one value of *s* (e.g., the weight average *s* value). This method for determination of molecular weight distribution is useful for large glycoconjugates [9] and associative complexes of very large molecular weight which are out of reach of other methods of analysis.

Interactions can manifest themselves in the appearance of material of a higher sedimentation coefficient, and if the reaction is reversible (or partially reversible) an increase in the slope of a plot of sedimentation coefficient *s*_20,w_ *vs.* concentration, *c* (the normal trend—due to non-ideality effects—is a decrease). The proportion of higher molecular weight component(s) should increase as the concentration increases and *vice versa*.

## 4. Sedimentation Equilibrium

In sedimentation equilibrium we analyse the steady state distribution of solute concentration *c(r)* as a function of radial displacement *r* from the centre of rotation to obtain an estimate for the molecular weight. For an interacting system it can in principle tell us the stoichiometry of the system, the average molecular weight of all the components in the solution being analysed (principally the weight average *M_w_* but also z-average *M_z_* and number average information *M_n_*) and also how the local or “point” average values *M_w_(r)* (and also *M_z_(r)* and *M_n_(r)*) vary with *r* and *c(r)*. From this, and the help of various diagnostic plots it is possible also to ascertain association/interaction *K_a_* or dissociation *K_d_* constants (often expressed in μM^−1^ or μM respectively) from the way the molecular weight changes with concentration. A popular algorithm in the past has been MSTAR based on the M* function of Creeth and Harding [10] and this has very recently been incorporated into SEDFIT as the very easy to use SEDFIT-MSTAR algorithm [11] which provides an estimate for *M_w_*, *M_w_(r) vs.* *r* or *c(r)* and through a smart-smooth procedure an estimate for the molecular weight distribution *c(M) vs.* *M*. Except at very small concentrations (<1 mg/mL for proteins, <0.3 mg/mL for large glycoconjugates) thermodynamic non-ideality (co-exclusion and polyelectrolyte effects) can be significant—estimates will then be of the *apparent* molecular weight (e.g., *M_w,app_*).

An associated algorithm MULTISIG [12] provides accurate estimates of *M_n_(r)* *vs.* *c(r)*, *M_z_(r)* *vs.* *c(r)* as well as *M_w_(r)* *vs.* *c(r)* and is proving very useful in the diagnosis of reversibility in interaction processes [13,14].

For non-interacting systems the conventional way of correcting for non-ideality is to measure *M_w,app_* at a series of concentrations and extrapolate back to zero concentration. Unfortunately if reversible concentration dependent interactive effects are being studied, this procedure is inappropriate. However the extent of non-ideality (as manifested by the 2nd thermodynamic virial coefficient B) can be predicted if the molecular weight and shape of the interacting species are known using the excluded volume theory of Rallison and Harding [15] and incorporated into the COVOL algorithm [16]: examples were demonstrated in the Harding and Rowe [1] review.

## 5. Protein Self-Association

Recent examples include the application of AUC to the analysis of the self-association of the tetanus toxoid protein and agrin.

### 5.1. Tetanus Toxoid Protein

**Figure 1:**
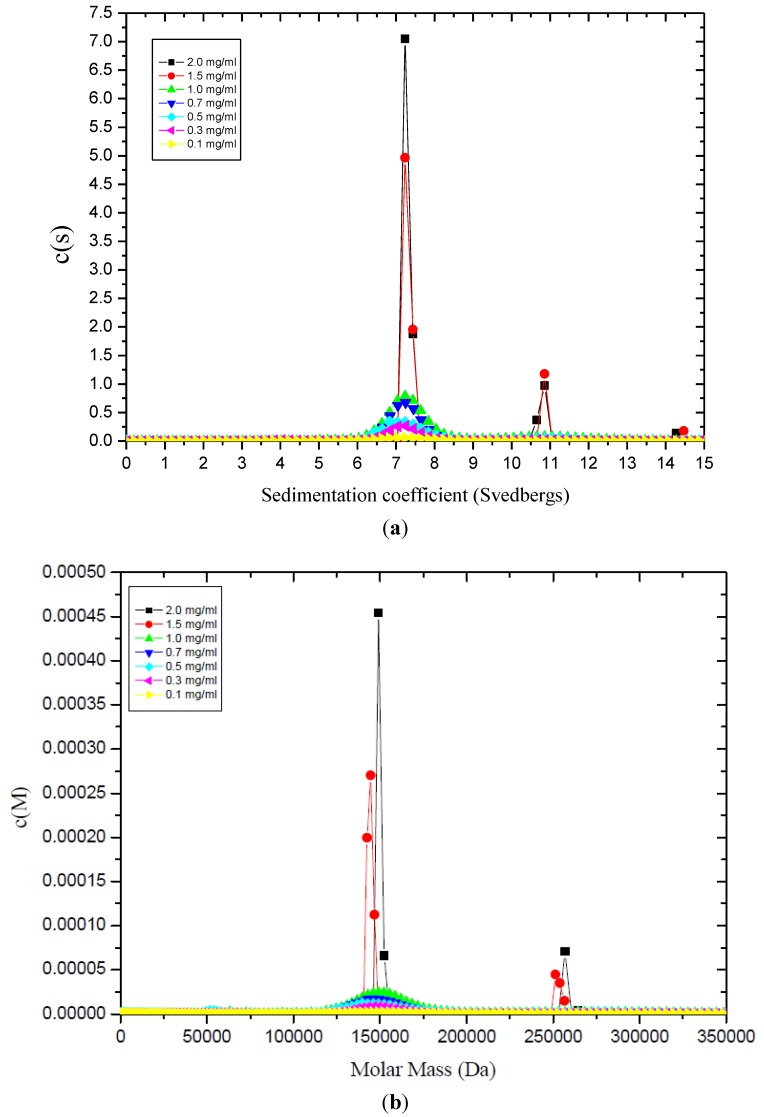

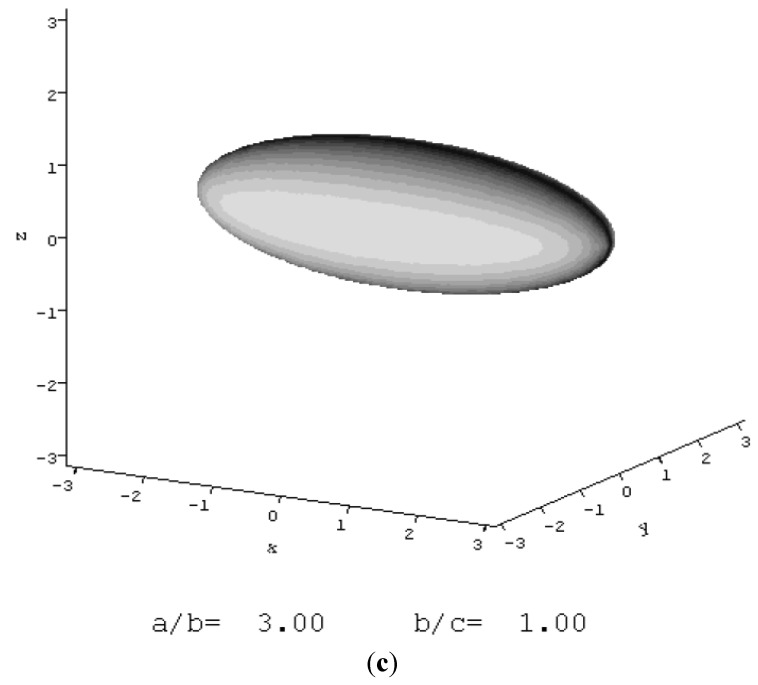
(**a**) Sedimentation coefficient distribution profiles, c*(s)* *vs.* *s*, for tetanus toxoid protein at different concentrations, showing mostly monomer with a smaller proportion of dimer not in reversible equilibrium; (**b**) Corresponding molecular weight distribution plots *c(M)* *vs.* *M*; (**c**) Low resolution structure of tetanus toxoid protein using the simple ellipsoid modelling routine ELLIPS1 showing clearly its extended form of axial ratio ~3:1. Reproduced from reference 18 with permission from Elsevier.

The tetanus toxoid (TT) protein is a chemically detoxified form of the tetanus toxin produced from *Clostridium tetani*. The tetanus neurotoxin is a 1292 amino acid protein consisting of two chains (N-terminal light chain of 52 kDa and C-terminal heavy chain of 98 kDa) linked by a single disulfide bridge. In addition to being a potent antigen, tetanus toxoid has been frequently used as the carrier protein in conjugate vaccines (see for example [17]), adding much greater efficacy to carbohydrate vaccines (see, e.g., [18]). Sedimentation coefficient *c(s)* *vs.* *s* profiles (Figure 1a) revealed the presence of two clear components with approximately 86% monomer sedimenting at ~7S and a 2nd component at ~11S (expected for the dimer, if *s ~ M^2/3^*). The proportions of each do not seem to alter significantly with increasing total loading concentration (Figure 1a): this implies that these two components are not in reversible equilibrium. Extrapolation of *s_20,w_* values of both components to *c* = 0 yielded *s°_20,w_* values of (7.6 ± 0.1)S and (11.6 ± 0.2)S for the two components. The corresponding *c(M)* *vs.* *M* plots (Figure 1b) yielded molecular weight values of ~(150,000 ± 5000) g/mol for the main peak and (270,000 ± 15,000) g/mol for the minor peak, consistent (within experimental error) with a monomer-dimer system, results which were consistent with sedimentation equilibrium and SEC-MALS (size exclusion chromatography coupled with multi-angle light scattering). Furthermore, from the sedimentation coefficient for the monomer combined with the intrinsic viscosity, it was possible, after allowing for a range of plausible hydration values [18], to estimate the overall shape of the TT protein in terms of a prolate ellipsoid, and the measurements are consistent with an asymmetric protein of aspect ratio ~(3 ± 1):1 (Figure 1c), coincidentally similar to the cartoon representation of it presented some years earlier by Astronomo and Burton [17]. Such a structure presents a greater surface area for conjugation with polysaccharide than a more globular structure, underpinning its popular choice as a conjugation protein for glycoconjugate vaccines.

### 5.2. Yeast Exoribonuclease Rrp6 Associated Cofactor Rrp47

In contrast to the TT-protein, recent measurements [19] using both sedimentation velocity and sedimentation equilibrium have established that the protein Rrp47, which plays an important part in RNA processing and degradation events, is a pure dimer at least under the conditions analysed, with an *s* value of (2.7 ± 0.1)S and M ~ 54,000 g/mol, but like TT-protein it has large frictional ratio (~1.6) corresponding to an axial ratio of ~6:1 [19].

### 5.3. Mini-Agrin

Mini-agrin [20] is a miniaturized form of agrin—an extracellular matrix proteoglycan that induces acetylcholine receptor aggregation at the neuromuscular junction, miniaturized so that it just contains 4-domains, the N-terminal domain and 3 globular C-terminal domains, considered to be potentially important in the treatment of congenital muscular dystrophy. Unlike the tetanus toxoid system sedimentation velocity in the analytical ultracentrifuge showed no direct evidence of associative behaviour from the *c(s)* *vs.* *s* plots but the weight average *s*°*_20,w_* value (4.7 ± 0.2)S combined with an estimate from dynamic light scattering for the translational diffusion coefficient *D*°*_20,w_* ~ (3.3 ± 0.2) × 10^−7^ cm^2^/s yielded an estimate of the *M* of (127,000 ± 12,000) g/mol using the Svedberg equation, well in excess of the sequence monomer molecular weight of 106,650 g/mol suggestive of associative behaviour. This was confirmed by SEC-MALS which revealed a value for the 2nd virial coefficient *B* of −6.5 × 10^−4^ mL·mol·g^−2^. On the grounds that return of a negative value for *B* signifies that thermodynamic non-ideality in the classical sense (exclusion volume and polyelectrolyte effects) is +ve and we observe –ve values confirms that self-association is significant. Re-analysis of the sedimentation equilibrium data using a routine which specifically considers the evaluation of the association/dissociation constants yielded and estimate of 0.24 L/g for the association constant—corresponds to a molar dimerization constant *K*_d_ ~ (4.2 ± 0.4) μM.

The low value for the sedimentation coefficient for such a large ~100 kDa protein suggests the agrin monomer is asymmetric with an axial ratio ~3.8, coincidentally similar to the tetanus toxoid protein. Further experiments using small angle x-ray scattering and modelling the angular distributions of scattered x-rays reinforced this view and indicates dimerization via an end-to-end rather than a side-by-side process [20].

## 6. Carbohydrate Self-Association

Certain classes of carbohydrate polymer are known to form double or triple helices (for example xanthan and schizophyllan respectively) but these tend to be non-reversible equilibria, *i.e.*, by “reversible” we mean by changing the concentration one can reversibly change the relative amount of oligomerization. Two systems have been shown to give reversible behaviour—one a very weak dimerization (xyloglucan) the other a stronger oligomerisation (aminocelluloses).

### 6.1. Xyloglucans

A very weak reversible dimerization was observed for xyloglucans—these are β(1–4) linked xylose polymers with arabinose (sometimes esterified with phenolic acids) side chains [21]. Evidence for dimerization comes not from the appearance of an extra component in the sedimentation coefficient distribution plots but from the concentration dependence of the sedimentation coefficient after allowing for non-ideality. For example one xyloglucan (PO2) gave a dissociation constant value *K*_d_ ~ (340 ± 50) μM at 20 °C. Intriguingly decreasing the temperature to 5 °C greatly suppressed the interaction(*K*_d_ > 3000 μM) whereas raising the temperature to 30 °C increased the dimerization strength (*K*_d_ ~ 140 μM): this decrease in *K*_d_ (increase in dimerization) with increase in temperature is systematic of a reversible hydrophobic interaction [21].

### 6.2. Aminocelluloses

**Figure 2:**
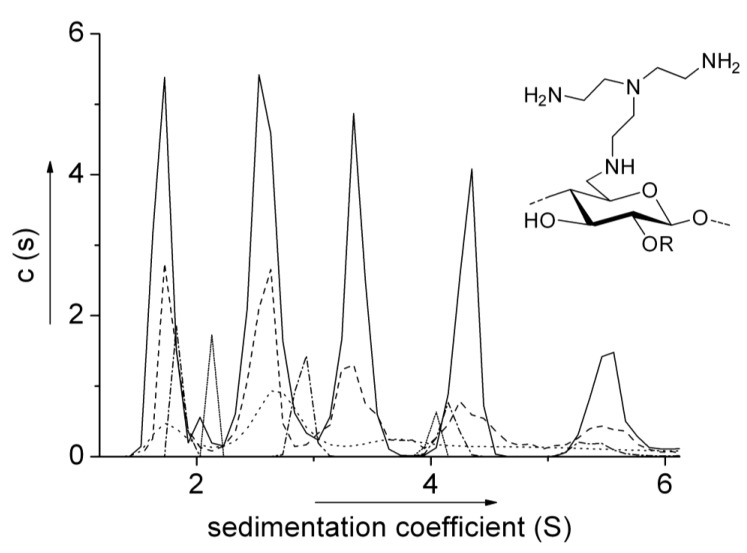
Sedimentation coefficient distributions of a 6-deoxy-6-amino cellulose (BAEA cellulose) at various concentrations: solid (──) 2.0 mg/mL; dash (– –) 1.0 mg/mL; dot (∙∙∙∙∙∙) 0.5 mg/mL; dash dot (– ∙ – ∙) 0.25 mg/mL; short dot (∙∙∙∙∙∙) 0.125 mg/mL. Reproduced from [13] with permission from Wiley.

Even more unusual *protein-like* association has been observed in solutions of the water soluble carbohydrates known as the 6-deoxy-6-(ω-aminoalkyl) aminocelluloses which had previously been reported to produce controllable self-assembling films for enzyme immobilisation and other applications [22]. *c(s) vs.* *s* distributions show multiple components (up to pentamers) all related according to *s* ~ *M* ^2/3^ (Figure 2). Not only had such oligomerisation—of the sort seen for example in antibody aggregation processes—never been seen in polysaccharides, the power law coefficient of 2/3 is more appropriate for globular proteins rather than carbohydrates [13]. The oligomerisation was at least partially reversible (the proportion of the lower molecular weight components decreasing as the total concentration is increased). One particular aminocellulose exhibited a completely reversible tetramerisation behaviour [14] as deduced on the basis of (i) point average molecular weights, *M_n_(r)*, *M_w_(r)* and *M_z_(r)* all converging to the same (monomer value) *M*_1_ = 3250 at zero concentration (and converging to the tetramer value at higher concentration) in plots of these point averages *vs.* *c(r)* using the MULTISIG routine and (ii) overlap of plots of *M_z_(r) vs.* *c(r)* for different loading concentrations. This completely reversible tetramerisation (and subsequent assembly into larger structures) resembled more like the behaviour of sickle cell deoxyhemoglobin than any carbohydrate, possibly revising traditional views of what is “protein-like” and what is “carbohydrate-like” behaviour (see [14] and references therein).

## 7. Carbohydrate-Protein Interactions

**Figure 3:**
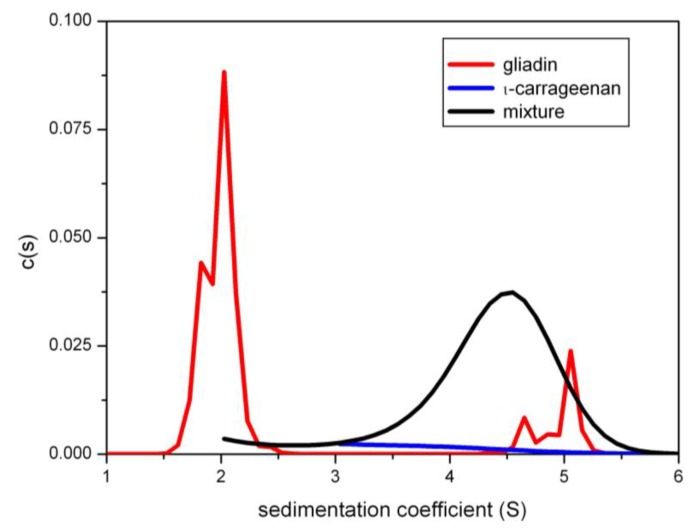
Sedimentation coefficient distribution diagrams for gliadins and iota carrageenan in aqueous phosphate-chloride buffer. *c(s)* = the population of species with a sedimentation coefficient between *s* and d*s*. UV-absorption optics at 280 nm were used showing only the gliadins—and whatever they may have interacted with. Red line: gliadin only control at 5.0 mg/mL loading concentration showing material sedimenting at 2S and a small amount of aggregated material at ~5S. Blue line: iota- carrageenan control at 1.0 mg/mL. The sedimenting material is almost transparent at 280 nm. Black line (same concentrations): mixture showing a substantial amount of material sedimenting at ~4.5S: this may indicate an interaction with gliadin. Reproduced with permission from [23].

Recent work on carbohydrate-protein interactions has been focusing on the possible use of fibre polysaccharides as a macromolecular block trying to stop gliadins reaching the mucosal epithelia and causing an immune response in people with gluten intolerance or coeliac disease. The work is currently on going, but an assay procedure based on sedimentation velocity in the analytical ultracentrifuge monitoring for co-sedimentation has been developed. The assay procedure takes advantage of the fact that gliadins absorb ultraviolet light at a wavelength ~280 nm whereas polysaccharides do not. For example iota-carrageenan [23]. Figure 3 shows sedimentation concentration distribution plots for a co-sedimentation experiment involving a mixture of gliadin and iota-carrageenan, showing a significant amount of what appears to be complexed material picked up by the UV absorption system at 280 nm and sedimenting at ~4.5S, well in excess of unbound gliadin. A comprehensive survey of fibre polysaccharides as potential macromolecular barriers to gliadin will be published in the near future (see also ref. [24]).

## 8. Nucleic Acid-Carbohydrate Interactions

There is increasing interest in the use of polycationic polysaccharides as histone analogues for condensing nucleic acids in DNA/RNA therapies against disease. Chitosans have been the focus of particular attention: these are soluble derivatives of chitin (poly N-acetyl glucosamine). Reducing the degree of acetylation (DA) yields the soluble polycationic form of chitin known as chitosan. In a recent study [25] (Almutairi *et al.*, 2014), the principle of co-sedimentation was used to explore the effectiveness of chitosans for binding to DNA, in a similar way to earlier studies applied to the study of chitosan “mucoadhesive” types of interaction with mucins [1,2,3]. In the Almutairi *et al.* study, solutions of two chitosans samples of different degrees of acetylation, known as “CHIT5” (DA = 25%) and “CHIT6” (15%) and different weight average molecular weights *M_w_* (95,000 g/mol and 170,000 g/mol respectively) from sedimentation equilibrium were characterised and then studied in mixtures with the DNA of *M_w_*, estimated from the *Extended Fujita* method of Harding *et al.* [8] to be approximately 300,000 g/mol.

**Figure 4:**
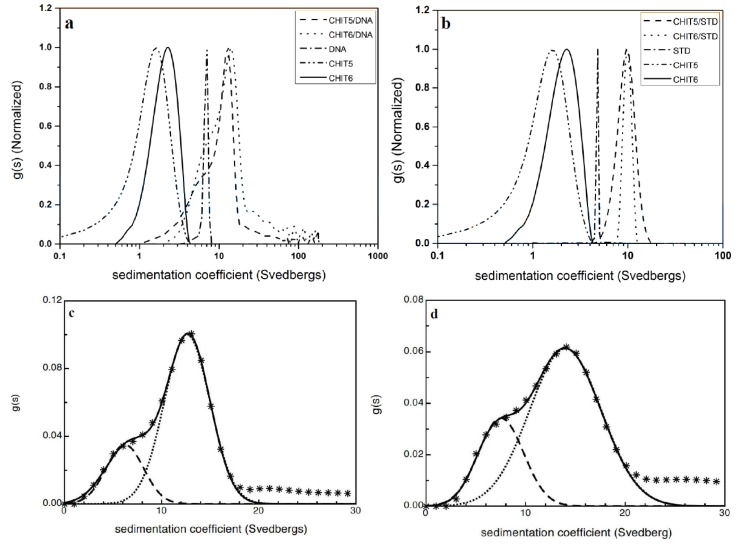
Normalized sedimentation coefficient distribution profiles obtained from sedimentation velocity experiments for unmixed controls and mixtures (**a**) chitosan with DNA; (**b**) chitosan with xanthan-STD; (**c**) multi-Gaussian fit to the CHIT5-DNA mixture; (**d**) multi-Gaussian fit to the CHIT6-DNA mixture. Reproduced from [25] with permission from Elsevier.

Sedimentation velocity of 1.0 mg/mL solutions of the chitosans CHIT5 or CHIT6 with DNA at a temperature of 20.0 °C all gave unimodal distributions with respective weight average sedimentation coefficients *s_20,w_* 1.9S, 2.3S and 6.8S respectively. Each chitosan preparation was then mixed in a 1:1 w/w ratio with the DNA to a total concentration of 1.0 mg/mL. A clear shift to a high *s* value is observed in both cases, with nothing sedimenting at the rate of uncomplexed chitosan suggesting that the interaction had gone to completion. From the shoulder of the complex, some unreacted DNA appears remaining (Figure 4a). Multi-Gaussian analysis of the *g(s)* *vs.* *s* distribution for the complex (Figure 4c) using the routine MULTIG suggests ~72% DNA had interacted with CHIT5 and ~83% for CHIT6. The *s*-distribution of the complex is very broad, even on a logarithmic scale, with material in excess of 100S, with the larger molecular weight and highest positively charged chitosan CHIT6 showing the greatest degree of complexation [4]. For a more detailed quantification of the nature of the interaction, other factors such as the differences in partial specific volume between DNA and carbohydrate would need to be taken into consideration.

## 9. Carbohydrate-Carbohydrate Interactions

### 9.1. Chitosan-Xanthan Interactions

The advantageous properties of mixtures of chitosan and xanthan as hydrogels have been considered for some while [26,27,28] but only very recently has the biophysical basis behind this interaction been studied by analytical ultracentrifugation, again using co-sedimentation [25]. Xanthan, like DNA is a double helical highly polyanionic polymer obtained from the microorganism *Xanthomonas campestris*, and is widely used in the Food Industry as a thickener. The molecular weight of the particular xanthan used in the study of Almutairi *et al.* [25]—known as “xanthan-STD”—had been found previously to be ~3.2 × 10^3^ kDa using sedimentation equilibrium in the analytical ultracentrifuge [29].

As with the chitosan-DNA study, the sedimentation velocity profile of xanthan was first characterised at 1.0 mg/mL yielding a unimodal *g(s)* *vs.* *s* plot with weighted-average *s_20,w_* of 4.8S. Then the 1:1 by weight mixtures with CHIT5 and CHIT6 at a total concentration of 1.0 mg/mL, were studied and the results shown in Figure 4b,d. As with chitosan-DNA, all the chitosan was complexed, but unlike chitosan-DNA, for xanthan-DNA there was no residual xanthan were left behind for both CHIT5 and CHIT6 complexes. Weighted average *s_20,w_* for the complexes of 10.3S and 10.1S for CHIT5-xanthan and CHIT6-xanthan, respectively were obtained.

### 9.2. PGX^®^

Sedimentation coefficient distribution profiling has also been used to good effect for detecting interactions in mixed polysaccharide systems used for health applications. An example is the proprietary commercial product PGX^®^ (PolyGlycopleX^®^) which is manufactured from konjac glucomannan, xanthan and sodium alginate using a proprietary manufacturing process (EnviroSimplex^®^, Inovobiologic inc. Burnaby, Canada). This product has been successfully used as a dietary/satiety product for help towards obesity control. Detailed analysis by co-sedimentation [30,31] showed clear evidence for a ternary interaction between the components at low ionic strength reinforcing experiments on the rheological properties of this system [32]. Interactions were shown to be clearly ionic strength dependent, clearly illustrating the importance of the solvent environment in the behaviour of polyelectrolyte polymers in solution.

## 10. Concluding Remarks

In our earlier review [1], we showed how powerful the analytical ultracentrifuge—with its intrinsic separation and analysis ability without the need for a separation matrix—was for characterising the strength, stoichiometry and reversibility for interacting protein (or glycoprotein) systems, and how potentially complicating issues such as thermodynamic non-ideality could be adequately dealt with.

In the present study, we have shown the considerable diversity of systems that the two main AUC techniques of sedimentation velocity and sedimentation equilibrium can be applied to with examples from very recently published studies, particularly using the principle of co-sedimentation. Particularly exciting is the application of the method for scrutinising the stability and behaviour of antibody based therapies—where the method is already considered as a gold standard—and also glyco-vaccine and DNA based therapy systems and the design of macromolecular based therapies against allergies and obesity. Other exciting developments include the improvement of ways of using and applying the analytical ultracentrifuge to high concentration systems, as is important for example in the development of monoclonal antibody formulations. This can provide a highly crowded macromolecular environment with a concomittant situation of high nonideality, well beyond the dilute solution situations considered in refs [15] and [16]. The use of tracer preparative (as opposed to *analytical*) ultracentrifugation in this regard, as pioneered by Minton, Rivas and others [33] has now been extended to centrifugal separation followed by subsequent analysis by solid state NMR ([34,35] and references therein).

For details of some of the latest developments and publications in the application of the analytical ultracentrifuge to the study of macromolecular interaction phenomena the interested reader is advised to refer to the web pages (and links given therein) of the National Centre for Macromolecular Hydrodynamics (www.nottingham.ac.uk/ncmh) and that of the SEDFIT user group: www.analyticalultracentrifugation.com/.
